# Comparison of laboratory-developed methods for aztreonam plus ceftazidime-avibactam antimicrobial susceptibility testing for metallo-beta-lactamase-producing Enterobacterales

**DOI:** 10.1128/jcm.01800-25

**Published:** 2026-05-22

**Authors:** Amelia S. Bhatnagar, Harley Harris, Emily Jacobs, Porscha Bumpus-White, Rocío Balbuena, Haley Stambaugh, Karlos Crayton, Jennifer Haynie, Justina Quintero Ilutsik, Cynthia Longo, Yehudit Bergman, Sweety Singh, Meghan Murray, Sarah Sabour, Matthew L. Robinson, María-José Machado, Patricia J. Simner

**Affiliations:** 1 Division of Healthcare Quality Promotion, Centers for Disease Control and Prevention 1242, Atlanta, Georgia, USA; 2 Department of Pathology, Division of Medical Microbiology, Johns Hopkins University School of Medicine 1500 , Baltimore, Maryland, USA; 3 Goldbelt C6, Juneau, Alaska, USA; 4 Department of Medicine, Division of Infectious Diseases, Johns Hopkins University School of Medicine 1500 , Baltimore, Maryland, USA; 5 BJ Government Medical College-Johns Hopkins Clinical Research Site 29593 , Pune, India; 6 Johns Hopkins Center for Infectious Diseases in India, Pune, India; 7 Department of Laboratory Medicine and Pathology, Division of Clinical Microbiology, Mayo Clinic 6915, Rochester, Minnesota, USA; Cleveland Clinic, Cleveland, Ohio, USA

**Keywords:** antimicrobial susceptibility testing, metallo-beta-lactamases, aztreonam-avibactam, synergy testing

## Abstract

Metallo-beta-lactamase (MBL)-producing Enterobacterales are a global public health concern. Infections caused by these pathogens are often hard to treat, with few effective agents currently available. While aztreonam-avibactam only recently gained U.S. and European regulatory approval, laboratories have implemented laboratory-developed tests (LDTs) using existing aztreonam and ceftazidime-avibactam reagents. We sought to compare the performance of four phenotypic methods, up to three reagent manufacturers, and two media manufacturers. One hundred two MBL-producing Enterobacterales isolates were evaluated at two sites (Centers for Disease Control and Prevention [CDC] and Johns Hopkins University [JHU] School of Medicine). The performance of four methods was evaluated: broth disk elution (BDE), disk stacking (DS), gradient diffusion strip stacking (SS), and a strip-to-disk proximity (strip-disk) method, which were compared to reference broth microdilution (rBMD) tested in parallel. Respective category agreement for CDC and JHU was 92.2% and 96.1% for BDE, 51%–76.5% and 31%–49% for DS, 71.6%–86.3% and 83.3%–86.3% for SS, and 82.4%–87.3% and 80%–89.2% for the strip-disk method. The SS essential agreement ranged from 46.1% to 62.7% for JHU and 54.9% to 92.2% for CDC. Interlaboratory reproducibility was 93.1% for rBMD, 92.2% for BDE, 47.1%–53.1% for DS, 76.5%–80% for SS, and 87.3%–88.9% for strip-disk. BDE was the only method that exceeded acceptable performance of >90% accuracy and reproducibility. DS had the lowest and most variable performance between sites and among the different disk manufacturers used. LDTs for combination testing can be highly variable, and further work is needed to improve standardization and harmonization to achieve accurate and reliable results for clinical purposes.

**IMPORTANCE:**

Carbapenem-resistant Enterobacterales are classified as an urgent public health threat in the Centers for Disease Control and Prevention’s 2019 Antibiotic Resistance Threats Report. Among the most concerning resistance mechanisms are carbapenemase enzymes encoded on mobile genetic elements, which can spread rapidly between bacteria. Of the various carbapenemases, metallo-beta-lactamases are particularly challenging, as they confer resistance to nearly all beta-lactams, and current treatment options remain limited. While aztreonam-avibactam has been U.S. Food and Drug Administration (FDA)-approved for treating certain infections with limited or no alternative treatment options, no FDA-cleared susceptibility test is yet available. In this context, laboratory-developed methods, such as combination testing of aztreonam and ceftazidime-avibactam, play a critical role in guiding therapy. Our study evaluates several of the more common laboratory-developed tests compared to reference broth microdilution and found that broth disk elution was the most accurate; in contrast, other methods had variable performance, some demonstrating limited reliability and being prone to overcalling resistance.

## INTRODUCTION

Metallo-beta-lactamase (MBL)-producing Enterobacterales are a global public health concern (1, 2). MBLs are a type of carbapenemase that confers resistance to a broad range of beta-lactam drugs, including carbapenems. Consequently, infections caused by these pathogens are often hard to treat due to the lack of available effective treatment options and lack of agents in development targeted toward these organisms (3).

Aztreonam, a monobactam, is stable in the presence of MBLs; however, it is vulnerable to hydrolysis by Ambler class A and C beta-lactamases, which are often concomitantly produced along with MBLs (4, 5). Avibactam, a non-beta-lactam-beta-lactamase inhibitor, counteracts these other non-MBL-beta-lactamases (6), thereby restoring aztreonam’s activity. Aztreonam-avibactam (AZA) received approval by the U.S. Food and Drug Administration (FDA) in February 2025 and in April 2024 by the European Medicines Agency and European Commission for marketing and for the treatment of certain infections caused by gram-negative bacteria when other treatment options are limited. This therapy is effective against infections caused by MBL-producing Enterobacterales. However, despite regulatory approval of AZA as a therapeutic, no FDA-cleared or Conformité Européenne (CE)-marked *in vitro* diagnostic for AZA susceptibility existed at the time of writing; however, after the study period, few tests have recently obtained FDA clearance or CE marking. Despite few tests becoming available, laboratories have and continue to use validated laboratory-developed antimicrobial susceptibility testing methods to assess aztreonam (ATM) and ceftazidime-avibactam (CZA) (ATM-CZA) susceptibility for highly resistant clinical isolates; these methods include utilizing gradient diffusion strips and either crossing the strips at designated minimum inhibitory concentrations (MICs) or stacking the strips on top of each other (7), utilizing antibiotic disks by placing them a distance from each other (disk proximity method), stacking disks, placing them in broth, or placing a disk next to a gradient diffusion strip (8, 9), checkerboard antimicrobial susceptibility testing, time-kill studies (10), or reference broth microdilution (rBMD).

The United States Centers for Disease Control and Prevention (CDC)’s Global Action in Healthcare Network-Antimicrobial Resistance Module (GAIHN-AR), a part of CDC’s broader Global Antimicrobial Resistance Laboratory and Response Network, is a public health initiative focusing on detecting and containing carbapenemase-producing Enterobacterales in healthcare settings through coordinated laboratory and infection prevention communication and strategies (11). GAIHN-AR comprises four implementing partners across five countries: India, Greece, Argentina, Chile, and Ethiopia. Given the global concern, and particularly for India due to a high prevalence of MBL-producing Enterobacterales, there was considerable interest to identify reliable and accessible methods for susceptibility to ATM-CZA. Furthermore, aside from reference broth microdilution and time-kill studies, which are also complex and resource-intensive, ATM-CZA methods lack clear standardization; therefore, studies evaluating these methods often have small differences in methodology, preventing standardized literature comparisons. Given the absence of standardized clinical guidelines for most of these methods, our goal was to compare their performance directly with rBMD and assess which method(s) could reliably support clinical decision-making in treating infections caused by MBL-producing Enterobacterales with the combination ATM plus CZA, or alternatively for AZA in the absence of an AZA-specific AST device.

To accomplish this, we sought to evaluate the *in vitro* performance of four practical, laboratory-developed methods to assess *in vitro* activity of ATM-CZA—broth disk elution (BDE), gradient diffusion strip stacking (SS), disk stacking (DS), and a strip-to-disk synergy (strip-disk) method—using multiple commercially available reagent manufacturers, particularly those available to the GAIHN-AR network. In doing so, we also aimed to provide standardized guidance for ATM-CZA susceptibility testing, which could be applied in India and elsewhere, including across GAIHN-AR.

## MATERIALS AND METHODS

### Bacterial isolates

A total of 102 contemporary de-identified MBL-producing Enterobacterales isolates from CDC’s collection (*n* = 47), Johns Hopkins University (JHU) School of Medicine Division of Medical Microbiology laboratory collection (*n* = 28), and CDC & FDA’s Antimicrobial Resistance Isolate Bank (AR Isolate Bank) (*n* = 27) (12) were selected for this study (Table S1). All isolates harbored at least an MBL gene confirmed by whole genome sequencing or an in-house real-time PCR for *bla*_NDM,_ *bla*_IMP,_ and *bla*_VIM._ Isolates were also characterized for other major carbapenemases, including *bla*_KPC_ and *bla*_OXA-48-like_. Two sites, CDC and JHU, used the same set of isolates for all four different testing methods.

### Methods

We assessed four phenotypic methods for ATM-CZA testing, including stacking gradient diffusion strips or disks, eluting aztreonam and ceftazidime-avibactam from disks in broth (broth disk elution), and placing a disk near a gradient diffusion strip to look for zone distortion. The comparator method used by both sites was reference broth microdilution.

#### Reference broth microdilution

At each site, the following methods were evaluated and compared to rBMD: BDE, gradient SS, gradient strip proximity (strip-disk), and DS. Reference broth microdilution panels were made according to CLSI M07 (13). The panel included the following drug concentration ranges (µg/mL): ATM (0.03125 - 64), CZA (0.03125/4 - 64/4), AZA (0.03125/4 - 64/4), and ATM-CZA (0.03125/0.3125/4 - 64/64/4). The cation-adjusted Mueller-Hinton broth (CAMHB) was made from Difco Mueller-Hinton broth base powder (BD, Franklin Lakes, NJ, USA), and the cation concentrations were adjusted to fall within CLSI requirements. Drug powders for aztreonam (TOKU-E, Bellingham, WA, USA), ceftazidime pentahydrate (TOKU-E, Bellingham, WA, USA), and avibactam sodium (Advanced ChemBlock, Inc., Hayward, CA, USA) were dissolved and diluted according to CLSI M100 (14). A single lot of rBMD panels was prepared by CDC and shared with JHU. rBMD AST was performed as previously described (15) following CLSI M07 guidelines. Briefly, isolates were suspended in sterile 0.85% saline, and the turbidity was adjusted to the equivalent of a 0.5 McFarland standard. The inoculum was added to 38 mL of sterile 0.85% saline, resulting in a 1:20 dilution. The rBMD panels were inoculated using 95-well pin inoculators (Caplugs, Dominguez, CA) and incubated at 35°C ± 2°C for 16 to 20 hours. rBMD panels were read manually against a black background using the unaided eye.

#### Method comparison design

**Fig 1:**
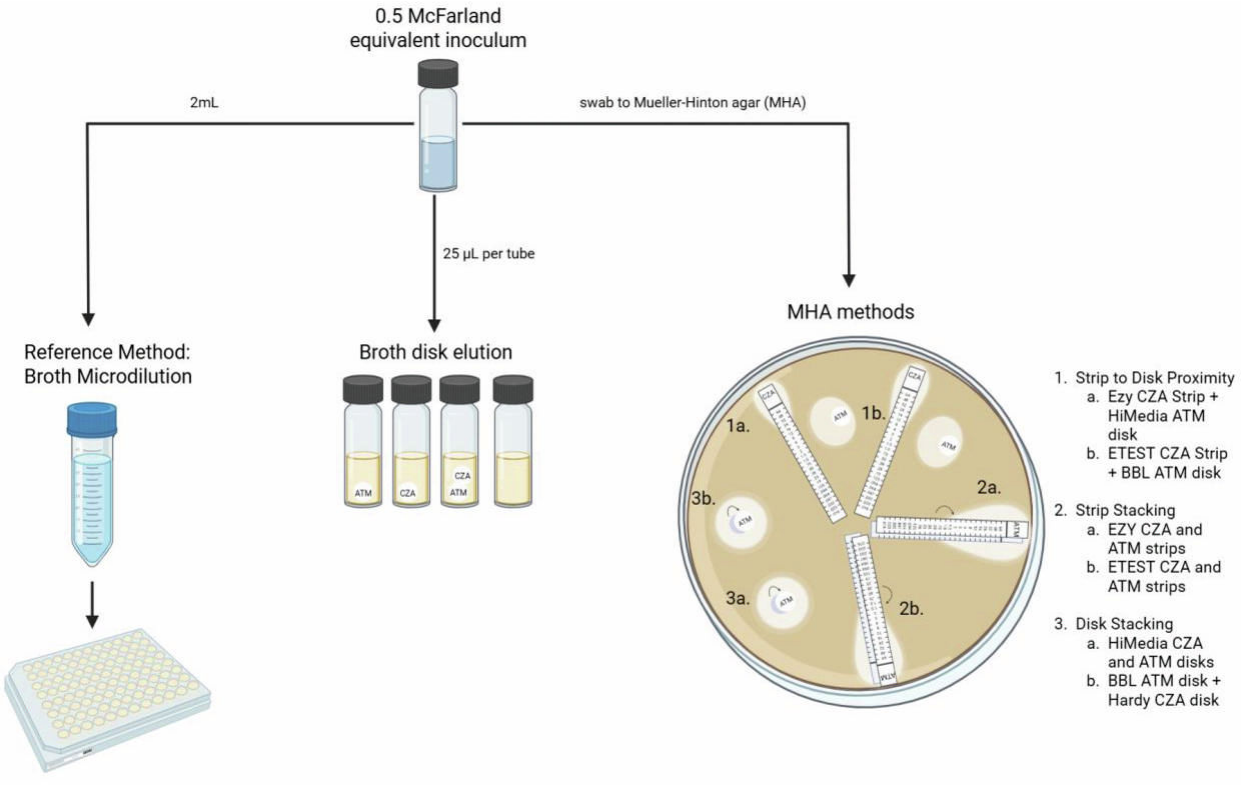
Schematic for testing five methods of antimicrobial susceptibility testing of ceftazidime-avibactam + aztreonam using the same inoculum. Figure 1 depicts the workflow for side-by-side testing. From the isolate inoculum equivalent to a 0.5 McFarland turbidity standard, 2 mL is transferred to a tube of 38 mL saline to proceed with the reference broth microdilution method (left arm). The second arm (middle) depicts the workflow for evaluating the broth disk elution method (single reagent manufacturers) by taking 25 µL from the inoculum and dispensing it into four tubes of 5 mL CAMHB. The third arm (right) depicts how the three methods and multiple reagent manufacturers, dependent on an inoculated MHA II agar, were set up on the same 150 mm plate. For JHU, a second plate was used to include the Liofilchem reagents. At each site, typically, there were staff who performed each arm of testing separately but in tandem, so that each step was completed within the appropriate time frame (e.g., using the inoculum within 15 minutes of preparation). Created in BioRender. Bhatnagar, A. (2025) https://BioRender.com/evo2kr0.

For SS, DS, and strip-disk methods, multiple reagent manufacturers were also tested (Table 1), including ATM (30 µg) and CZA (30/20 µg) disks from Hardy Diagnostics (Santa Maria, CA, USA), BD (Franklin Lakes, NJ, USA), and HiMedia (Mumbai, India), and the following gradient diffusion strips: ETEST (bioMérieux; Marcy-l'Étoile, France), MIC test strips (MTS) (Liofilchem; Roseto degli Abruzzi [TE], Italy), and Ezy MIC (HiMedia; Mumbai, India). All methods were performed in parallel on the same day. Frozen isolates were subcultured twice on trypticase soy agar with 5% sheep’s blood agar plates (BD BBL, BD, Franklin Lakes, NJ) and incubated at 35 ± 2°C in ambient air for 18–24 hours prior to susceptibility testing. From this plate, an inoculum suspension was made using three to five colonies adjusted to a 0.5 McFarland equivalent turbidity. The single inoculum was used across all tests (Fig. 1). At both sites, multiple laboratory personnel were included in the testing.

**TABLE 1:** Manufacturers and comparison combinations among methods evaluated at each site for aztreonam plus ceftazidime/avibactam testing^*a*^

Method or reagent	CDC	JHU
BDE	Hardy HardyDisks for ATM and BD BBL Sensi-discs for CZA	Hardy HardyDisks for ATM and BD BBL Sensi-discs for CZA
MHA II	Becton Dickinson BBL	Thermo Scientific Remel
Gradient diffusion SS	bioMérieux ETEST for ATM and CZA	bioMérieux ETEST for ATM and CZA
	HiMedia Ezy MIC for ATM and CZA	HiMedia Ezy MIC for ATM and CZA^*b*^
		Liofilchem MTS for ATM and CZA
DS	Hardy HardyDisks for ATM and BD BBL Sensi-discs CZA	Hardy HardyDisks for ATM and BD BBL Sensi-Discs CZA
	HiMedia disks for ATM and CZA	HiMedia disks for ATM and CZA^*b*^
		Liofilchem disks for ATM and CZA
Strip-to-disk proximity (strip-disk)	Hardy HardyDisk ATM and bioMérieux CZA ETEST	Hardy HardyDisks ATM and bioMérieux CZA ETEST
	HiMedia ATM disk and HiMedia CZA Ezy MIC	HiMedia ATM disk and HiMedia CZA Ezy MIC^*b*^
		Liofilchem ATM disk and Liofilchem CZA MTS

^*a*^ ATM, aztreonam; CZA, ceftazidime-avibactam.

^*b*^ JHU tested a subset of isolates with this manufacturer.

#### BDE

BDE was performed according to CLSI document M100 (14). Briefly, four prepared tubes of 5 mL CAMHB (BD, Franklin Lakes, NJ, USA) were used for each isolate. One ATM disk was added to the first tube, a CZA disk was added to the second tube, and both disks, ATM and CZA, were added to the third tube. The fourth tube was designated as a growth control; therefore, no disks were added. This resulted in the final concentrations (µg/mL) of ATM/CZA in each tube being 6/0/0, 0/6/4, 6/6/4, and 0/0/0, respectively. Any turbidity identified by the unaided eye in the tube was considered not susceptible (intermediate or resistant), while no turbidity was considered a susceptible result.

#### Agar-based methods: DS, SS, and strip-to-disk proximity

For DS, SS, and strip-disk methods, a Mueller Hinton II agar (MHA) plate was inoculated with the bacterial suspension. CDC used BD BBL MHA II agar, while JHU used Remel (Thermo Scientific, Waltham, MA, USA). CDC tested all methods on a single 150 mm MHA II plate. Because JHU tested three manufacturers of reagents, methods were performed across two 150 mm MHA II plates. Due to procurement timing (i.e., delay in acquisition), JHU tested HiMedia disks and gradient strips for the aforementioned MHA II-based methods for a subset of isolates (see Table 4 for subset numbers) in parallel with the rBMD method only.

For the DS, a CZA disk was placed on a quadrant of the inoculated MHA II plate. After 10 minutes, the CZA disk was removed, and at the same spot, replaced with an ATM disk (16). For the SS, a CZA gradient strip was placed on a quadrant of the inoculated MHA II plate. After 10 minutes, the CZA strip was removed, and at the same spot, replaced with an ATM strip (7). For strip-to-disk, a CZA strip was placed on a quadrant of the inoculated MHA II plate. An ATM disk was then placed 15 mm away (center of strip to center of disk) perpendicular to the 8/4 μg/mL concentration on the CZA strip (16).

After all methods were applied, the plate was incubated in ambient air at 35 ± 2°C for 16–18 hours. After incubation, the zone diameters of the DS and MIC of SS methods were measured and recorded. For the strip-disk method, a positive result was defined as any visible distortion of the zone of inhibition around the ATM disk toward the gradient diffusion strip, such as an inverse-D shape, whereas a negative result was defined as no distortion in the zone of inhibition around the ATM disk.

### Discrepancy testing

Repeat testing was conducted for very major and major errors (VMEs and MEs, respectively) compared to rBMD. Repeat testing was conducted by repeating the method(s) in error in parallel with rBMD. If a method and/or brand combination showed poor performance (category agreement [CA] of <75%) in the initial testing and required many repeats, the method was considered poor, and repeat testing was not performed.

### Quality control

For rBMD, ATCC *Escherichia coli* 25922, ATCC *Pseudomonas aeruginosa* 27853, and ATCC *Klebsiella pneumoniae* 700603 were used as control strains. For the BDE, ATCC 25922, BAA-1705, *K. pneumoniae* BAA-2146, and AR Isolate Bank’s *E. coli* AR-0348 were used. No standardized quality controls are available for ATM-CZA synergy methods; therefore, for the DS, SS, and strip-disk methods, an MBL-positive but ATM-CZA susceptible strain (BAA-2146) and an ATM-CZA resistant strain (AR-0348) were used as controls. Individual gradient diffusion strips and disks were tested for quality control each day of testing per manufacturer’s recommendations.

### Reproducibility testing

For intra-laboratory reproducibility, 10 separate dates of quality control testing were used to determine interlaboratory reproducibility. A single replicate was tested. For interlaboratory reproducibility, the sites independently selected a single date on which all isolates were tested three times from different inocula.

### Data analysis

Accuracy was demonstrated by calculating CA, MEs, VMEs, and minor errors (if applicable). The ATM-CZA result from the rBMD method was used as the comparator. FDA breakpoints for aztreonam-avibactam (susceptible = ≤4/4 µg/mL; intermediate = 8/4 µg/mL; resistant = ≥16/4 µg/mL) were used for ATM-CZA results unless otherwise specified (17). Essential agreement (EA), where the MIC of the test method was within ±1 twofold doubling dilution of the rBMD MIC result, was calculated for only the SS method, as this is the only method that produced an MIC result. The BDE and strip-disk methods only provide a not susceptible/positive or susceptible/negative result; therefore, minor errors were not assessed and inferred ATM-CZA breakpoints of ≥8/8/4 µg/mL for not susceptible (intermediate and resistant) and ≤4/4/4 µg/mL for susceptible were used. MIC bias was also assessed for SS using the International Organization for Standardization ISO 20776-1 method to determine the proportion of SS MICs that were at least one dilution higher and lower than rBMD MICs. A bias of ± ≥30% was considered indicative of a trend (18). Because not every method had repeat testing performed, the data presented are the initial data unless otherwise stated.

Interlaboratory reproducibility was assessed by comparing CDC and JHU test results for each method and manufacturer. AZA and ATM-CZA MIC biases were assessed by determining the difference in the proportion of ATM-CZA results that were at least one dilution higher or lower than the AZA MIC. We sought to determine whether the addition of a non-MBL carbapenemase (e.g., KPC or OXA-48-like) had any impact on the ATM-CZA MIC in either direction. Significant differences were determined by conducting a two-sample z-test for independent differences (*P* < 0.05) using R programming 4.5.0 (RStudio: Integrated Development Environment for R v.2024.12.1; Posit Software, Boston, MA).

We compared the performance between sites and among methods at a site using a χ^2^ test of significance (*P* < 0.05). For analyses involving more than two comparisons, the *post hoc* Tukey multiple comparison of proportions method was used to determine significant pairs (19). Statistical analyses were performed in Statistical Analysis Software (SAS 9.4 vTS1M8; SAS Institute, Cary, NC).

This activity was reviewed by CDC, deemed not research, and was conducted consistent with applicable federal law and CDC policy. See 45 C.F.R. part 46.102(l)(2), 21 C.F.R. part 56; 42 U.S.C. §241(d); 5 U.S.C. §552a; 44 U.S.C. §3501 et seq.

## RESULTS

### rBMD method

The organisms tested included the following genera/species: *Escherichia coli* (*n* = 37)*, Klebsiella pneumoniae* (*n* = 36), *Enterobacter cloacae* complex (*n* = 17), *Providencia rettgeri* (*n* = 4), *Morganella morganii* (*n* = 2), *Citrobacter freundii* (*n* = 2), *Enterobacter aerogenes* (*n* = 1), *Klebsiella oxytoca* (*n* = 1), *Salmonella enterica* serotype Senftenberg (*n* = 1), and *Citrobacter amalonaticus* (*n* = 1). The ATM-CZA MIC distribution among all species at both sites ranged from 0.06/0.06/4 to >64/64/4 µg/mL with an MIC_50_ of 2/2/4 µg/mL and an MIC_90_ of 16/16/4 µg/mL. The MIC distribution by site and genera (where the number of isolates ≥10) is provided in Table 2. We also compared the AZA MICs to the ATM-CZA MICs to assess whether the MICs with ceftazidime compared to AZA MICs were lower, as ceftazidime also has binding affinity for penicillin-binding protein 3 (Table 3). No significant differences were observed when comparing the MIC bias of isolates with only MBL present versus isolates that had an MBL plus another carbapenemase (e.g., OXA-48-like or KPC). Reproducibility of CDC and JHU ATM-CZA and AZA was 93.1%. Quality control was in range 97.9% of the time for CDC and 97.6% of the time for JHU.

**TABLE 2:** Summary of reference broth microdilution results for aztreonam plus ceftazidime-avibactam by site and organism genera

Organism	Site	*N*	*N* "S"	*N* "I"	*N* "R"	MIC range	MIC_50_	MIC_90_
All	JHU	102	76	3	23	0.06/0.06/4 – >64/64/4	2/2/4	16/16/4
	CDC	102	76	8	18	0.06/0.06/4 – >64/64/4	2/2/4	16/16/4
*Escherichia coli*	JHU	37	12	3	22	0.12/0.12/4 – >64/64/4	16/16/4	64/64/4
	CDC	37	12	8	17	0.12/0.12/4 – >64/64/4	8/8/4	32/32/4
*Klebsiella pneumoniae*	JHU	36	36	0	0	0.12/0.12/4 – 4/4/4	1/1/4	4/4/4
	CDC	36	36	0	0	0.12/0.12/4 – 4/4/4	1/1/4	4/4/4
*Enterobacter cloacae* complex	JHU	17	17	0	0	0.06/0.06/4 – 1/1/4	0.25/0.25/4	1/1/4
	CDC	17	17	0	0	0.06/0.06/4 – 2/2/4	0.25/0.25/4	1/1/4
Other Enterobacterales^*a*^	JHU	12	11	0	1	0.06/0.06/4 – >64/64/4	1/1/4	16/16/4
	CDC	12	11	0	1	0.12/0.12/4 – >64/64/4	1/1/4	16/16/4

^*a*^ Other Enterobacterales contained the following organisms: *Providencia rettgeri *(4), *Morganella morganii* (2), *Citrobacter freundii *(1), *Enterobacter aerogenes *(1), *Salmonella enterica *serotype Senftenberg (1),* Citrobacter amalonaticus *(1), and *Klebsiella oxytoca *(1)*. *S, susceptible; I, intermediate; R, resistant.

**TABLE 3:** Results of assessing the impact of ceftazidime in reference broth microdilution MICs of aztreonam plus ceftazidime-avibactam to MICs of aztreonam-avibactam

Category	Site	*N*	Number doubling dilutions of aztreonam + ceftazidime/avibactam MICs lower, equal, or higher than aztreonam/avibactam MICs					Percent in ±1^*a*^	Percent exact^*a*^	Percent ≤−1^*a*^	Percent ≥+1^*a*^	Bias^*a*^
			−2	−1	0 (Exact)	+1	+2					
All organisms	JHU	102	0	12	76	11	3	97.1%	74.5%	11.8%	13.7%	2.0%
	CDC	102	1	11	80	9	1	98.0%	78.4%	11.8%	9.8%	−2.0%
MBL alone	JHU	63	0	6	48	7	2	96.8%	76.2%	9.5%	14.3%	4.8%
	CDC	63	1	7	51	4	0	98.4%	81.0%	12.7%	6.3%	−6.3%
MBL + OXA or KPC	JHU	39	0	6	28	4	1	97.4%	71.8%	15.4%	12.8%	−2.6%
	CDC	39	0	4	29	5	1	97.4%	74.4%	10.3%	15.4%	5.1%
MBL + OXA	JHU	23	0	1	18	4	0					
	CDC	23	0	1	21	1	0					
MBL + KPC	JHU	16	0	5	10	0	1					
	CDC	16	0	3	8	4	1					

^*a*^ Only categories ≥30 datapoints were assessed.

### BDE method

For the BDE, both sites tested the same 102 isolates. Quality control was in range 97.2% of the time for CDC and 88.1% of the time for JHU (Table S3). Interlaboratory reproducibility between CDC and JHU was 92.2%. Both sites obtained CA >90% (Table 4) and were not significantly different. Both JHU and CDC had MEs and VMEs. CDC had two MEs and two VMEs, while JHU had six MEs and one VME. Of all errors, one ME was observed for the same isolate by both sites. For CDC, all errors occurred around the breakpoint used (4/4/4 for S and 8/8/4 for NS), while JHU had two of eight errors at the breakpoint. Repeat testing was conducted at both sites. Repeat testing resolved one ME for CDC; for JHU, repeat testing resolved three of eight errors, including the two (one VME and one ME) that occurred at the breakpoint (where the rBMD result changed from 4/4/4 µg/mL to 8/8/4 µg/mL, but the BDE result stayed the same) and an additional ME. Recalculated performance metrics using repeat testing results are available in Table S2.

**TABLE 4:** Performance of four phenotypic methods for aztreonam plus ceftazidime-avibactam AST compared to the reference broth microdilution result prior to repeat testing^*b*^

Method and brand	Reference broth microdilution results						Category agreement *n* (%)		Essential agreement *n* (%)		Minor errors *n* (%)		Major errors *n* (%)		Very major errors^*a*^ *n* (%)	
	JHU			CDC			JHU	CDC	JHU	CDC	JHU	CDC	JHU	CDC	JHU	CDC
	Total	*N* "S"	*N* "R"	Total	*N* "S"	*N* "R"										
**BDE**	102	76	26^*a*^	102	76	26^*a*^	94 (92.2)	98 (96.1)	NA	NA	NA	NA	6 (8.0)	2 (2.6)	2 (7.4)	2 (7.7)
**Disk stacking**																
Liofilchem	102	76	23	–	–	–	52 (51.0)	–	NA	NA	13 (12.7)	–	37 (48.7)	–	0 (0)	–
BBL/Hardy	102	76	23	102	76	18	78 (76.5)	50 (49.0)	NA	NA	20 (19.6)	24 (23.5)	4 (5.3)	28 (36.8)	0 (0)	0 (0)
HiMedia	49	26	21	102	76	18	34 (69.4)	32 (31.4)	NA	NA	9 (18.4)	20 (19.6)	6 (23.1)	50 (65.8)	0 (0)	0 (0)
**Strip stacking**																
Liofilchem MTS	102	76	23	–	–	–	73 (71.6)	–	47 (46.1)	–	18 (17.6)	–	11 (14.5)	–	0 (0)	–
bioMerieux ETEST	102	76	23	102	76	18	88 (86.3)	88 (86.3)	64 (62.7)	94 (92.2)	8 (7.8)	13 (12.7)	6 (7.9)	0 (0)	0 (0)	1 (5.6)
HiMedia Ezy MIC	50	27	21	102	76	18	40 (80.0)	85 (83.3)	31 (62.0)	56 (54.9)	9 (18.0)	14 (13.7)	0 (0)	2 (2.6)	1 (4.8)	1 (5.6)
**Strip-to-disk synergy**																
Liofilchem	102	76	26^*a*^	–	–	–	91 (89.2)	–	NA	NA	NA	NA	6 (7.9)	–	5 (21.7)	–
bioMerieux CZA ETEST + Hardy ATM	102	76	26^*a*^	102	76	26^*a*^	90 (88.2)	89 (87.3)	NA	NA	NA	NA	6 (7.9)	5 (6.6)	6 (23.1)	8 (30.8)
HiMedia	45	22	23^*a*^	102	76	26^*a*^	36 (80.0)	84 (82.4)	NA	NA	NA	NA	1 (4.5)	7 (9.2)	8 (34.8)	11 (42.3)

^*a*^ *N*(%); *BDE and strip-to-disk synergy methods considered not susceptible interpretations (intermediate or resistant) when calculating very major errors.

^*b*^ S, susceptible; R, resistant; ATM, aztreonam. JHU, John’s Hopkins University; CDC, Centers for Disease Control and Prevention; BDE, broth disk elution; MTS, MIC test strips; MIC, minimum inhibitory concentration; NA, not applicable; “–” indicates this method was not tested at the specified site.

### DS method

For disk stacking, JHU tested 102 isolates for both Liofilchem’s disks and HardyDisk (ATM)-SensiDisc (CZA)’s disks and 49 isolates for HiMedia disks; CDC tested 102 isolates for both HardyDisk-SensiDisc and HiMedia disks. Quality control was in range 88.2% of the time for HardyDisk-SensiDisc and 41.2% for HiMedia disks at CDC, and 79.1% of the time for Liofilchem and 100% of the time for both HardyDisk-SensiDisc and HiMedia for JHU (Table S3). The interlaboratory reproducibility between sites for HiMedia and HardyDisk-SensiDisc disks was 53.1% and 47.1%, respectively. The mean zone diameter difference between CDC and JHU was 4.6 and 4.8 mm for HardyDisk-SensiDisc and HiMedia, respectively.

Performance is summarized in Table 4. Briefly, for HardyDisk-SensiDisc disk combinations, CDC and JHU CA were 49% and 76.5%, respectively. Both sites observed high frequency (>10%) of minor errors. CDC had 28 (36.8%) MEs, while JHU had 4 (5.3%) MEs. Neither site observed any VME. All four MEs observed by JHU were also observed by CDC. For HiMedia, CDC’s and JHU’s CA were 31.4% and 69.4%, respectively. CDC observed 20 minor errors and 50 MEs, while JHU observed 9 minor errors and 6 MEs. When comparing the zone diameters of HardyDisk-SensiDisc and HiMedia disks, HiMedia disks tended to have smaller zones than HardyDisk-SensiDisc; at CDC, this frequency was 92.2% and at JHU, 100%. Only JHU performed testing for Liofilchem disks and observed 51% CA with 37 MEs and 13 minor errors. CDC did not perform repeat testing as the initial performance did not meet the aforementioned method repeat testing criteria. Due to the poor initial performance, JHU did not perform repeat testing for HiMedia or Liofilchem disks. The four JHU MEs from HardyDisk-SensiDisc disks were also observed when using HiMedia disks. Of the four MEs observed with the HardyDisk-SensiDisc disks, two of four errors were completely resolved, with one error repeating as a minor error. For JHU, Liofilchem CA was statistically significant from the CA of both HiMedia and HardyDisk-SensiDisc disks; HiMedia and HardyDisk-SensiDisc were not statistically significant from each other. Between sites, both manufacturers had CA that were statistically significant from each other. Recalculated performance metrics using repeat testing results are available in Table S2.

### SS method

For strip stacking, JHU tested 102 isolates for both MTS and ETEST strips and 50 isolates for Ezy MIC strips; CDC tested 102 isolates for both ETEST and Ezy MIC strips. Quality control was in range 100% of the time for both manufacturers at CDC, and for JHU, 100% of the time for both MTS and ETEST and 93.3% of the time for Ezy MIC (Table S3). The interlaboratory reproducibility between sites for ETEST strips was 76.5% and 80.0%, respectively.

Performance is summarized in Table 4. Briefly, for ETEST, the EA was 92.2% for CDC and 62.7% for JHU; CA (86.3%) was the same for both sites. The MIC bias for CDC was 24.9% and a 56.9% bias (and upward trend) was observed for JHU. CDC observed one VME and no MEs, whereas JHU observed six MEs and no VMEs. For JHU, five of six MEs were resolved, and for CDC, the one VME was also resolved. For Ezy MIC, the EA was 54.9% for CDC and 62.0% for JHU; CA was 83.3% for CDC and 80.0% for JHU. CDC observed an MIC bias and upward trend of 66.8%, while JHU observed a bias of 24.9%. Both sites observed a moderate number of minor errors. CDC had two MEs and one VME, while JHU had only one VME. For JHU, the single VME was reclassified as a minor error with repeat testing, and for CDC, one VME and one ME were resolved during repeat testing. For MTS, JHU observed an EA of 46.1%, CA of 71.6% with 11 MEs. Six of these errors were also the same isolates that caused MEs with ETEST. Due to the poor initial performance, JHU did not perform repeat testing for MTS. There were no significant differences in the CA among manufacturers tested nor between sites; however, there were significant differences in EA between sites for Ezy MIC, while MTS was significantly different from ETEST, but not Ezy MIC at JHU. Recalculated performance metrics using repeat testing results are available in Table S2.

### Strip-disk method

For the strip-disk method, JHU tested 102 isolates for both Liofilchem’s MTS/disk and HardyDisk ATM/ETEST CZA and 45 isolates for HiMedia’s Ezy MIC/disk; CDC tested 102 isolates for both HardyDisk ATM/ETEST CZA and HiMedia. Quality control was in range 100% of the time for HiMedia and 93.7% for HardyDisk ATM/ETEST CZA for CDC, and 97.7% of the time for Liofilchem, 100% of the time for HardyDisk ATM/ETEST CZA, and 50% of the time for HiMedia for JHU (Table S3). The interlaboratory reproducibility between sites was 87.3% for HardyDisk ATM/ETEST CZA and 88.9% for HiMedia.

Performance is summarized in Table 4. Briefly, for the HardyDisk ATM/ETEST CZA combination, JHU had a CA of 88.2% with six MEs and six VMEs, while CDC had a CA of 87.3%, five MEs, and eight VMEs. Six errors were common across the two sites. Repeat testing did not resolve any errors for JHU but did resolve two VMEs and one ME for CDC. For HiMedia, JHU had a CA of 80% with one ME and eight VMEs, while CDC had a CA of 82.4% with seven MEs and 11 VMEs. Seven errors were common across the two sites. Repeat testing at JHU resolved two VMEs, while CDC saw no resolution during repeat testing. For Liofilchem, JHU observed a CA of 89.2% with six MEs and five VMEs. Repeat testing resolved only one of six VMEs and did not resolve any MEs. There were no significant differences in performance between sites or among manufacturers. Recalculated performance metrics using repeat testing results are available in Table S2.

### Reproducibility testing

Intralaboratory reproducibility was acceptable (>95%) for all tests at both sites.

## DISCUSSION

In this study, we evaluated the performance of several methods for ATM-CZA susceptibility in comparison to reference broth microdilution—the gold standard method for antimicrobial susceptibility testing. Recently, a few manufacturers (Hardy HardyDisks, Thermo Scientific Oxoid disk, bioMérieux ETEST, and Liofilchem MTS) have commercially available tests; though, clinical laboratories have adopted various laboratory-developed methods to assess the potential effectiveness of the combination of ATM-CZA in anticipation of these commercially available tests. These approaches include qualitative methods (e.g., disk-to-disk synergy, disk-to-strip synergy [strip-disk], BDE) as well as quantitative methods (gradient diffusion SS, DS, agar supplementation) (7, 8, 16, 20–25). However, there is a lack of standardized guidance or widely adopted protocols or procedures for performing and interpreting these laboratory-developed tests. Our findings highlight variability in test performance, with the broth disk elution method demonstrating the highest accuracy, reproducibility, and consistency across two independent sites.

Among the methods evaluated, BDE demonstrated the highest accuracy, followed closely by the strip-disk method using either Liofilchem’s MTS and disk or ETEST CZA strip with a HardyDisk ATM, and then SS (all manufacturers). Several studies have also previously reported good performance for the BDE method when compared directly to reference broth microdilution (22, 23), including CLSI’s endorsement of the method in 2024. In contrast, DS had the lowest performance for CA, which has been reported by prior comparative studies (23, 26, 27). Khan et al. (23) evaluated BDE, DS, and SS on a small cohort of isolates (23) and despite some manufacturer, methodological, and analytical differences from our study, we observed similar results in that BDE and SS exhibited good performance, while disk stacking was less reliable. Although SS was somewhat effective in distinguishing between interpretive categories, the MIC agreement, evaluated by calculating EA, varied across manufacturers and sites. Overall, essential agreement across all manufacturers for SS was generally below 65% except for ETEST SS on BD BBL MHA II agar performed at CDC. Notably, CDC and JHU had a statistically significant difference in EA between the ETEST SS (92.2% versus 62.7%, respectively), raising concerns about the reproducibility of this method across different MHA II manufacturers—CDC used BD BBL and JHU used Remel—and laboratory conditions. In addition, significant differences in performance were observed between the JHU and CDC sites with DS. At CDC, the CA for DS was below 50% whereas JHU had CA above 75%. A substantial proportion of CDC discrepancies were due to ME, 65.8% for HiMedia and 36.8% for HardyDisk-SensiDisc, as well as numerous minor errors. One potential contributing factor is the use of different manufacturers of MHA II agar between sites, as different media manufacturers can have an impact on AST (28, 29). However, such differences in performance were not observed among all agar-dependent methods despite sites using different MHA II manufacturers.

**Fig 2:**
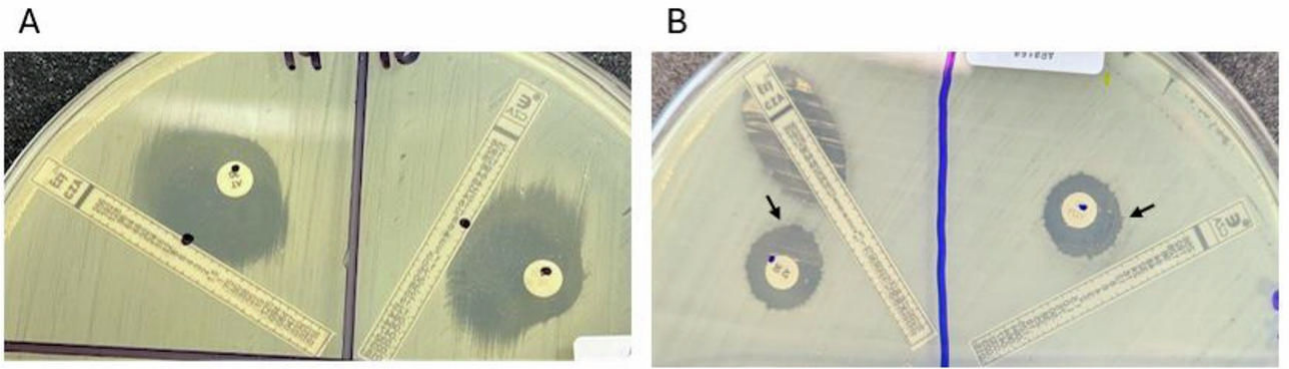
Obvious versus subtle zone distortion observed in the strip-to-disk proximity method for synergy of aztreonam + ceftazidime/avibactam. Strip-to-disk synergy-positive results for two different isolates. Panel **A** is AR0057 (*Morganella morganii*), where a clear distortion is seen of the aztreonam disk’s zone diameter toward the ceftazidime/avibactam gradient diffusion strip. Panel **B** is AR0150 (*Escherichia coli*) and depicts a more subtle deformation (arrow) of aztreonam disk’s zone diameter.

**Fig 3:**
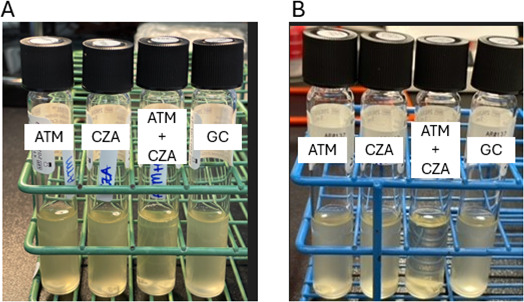
Light turbidity versus full turbidity observed in the broth disk elution method for testing of aztreonam (ATM) + ceftazidime-avibactam (CZA). Panel **A** depicts full turbidity of the tube containing ATM + CZA disks, while panel **B** depicts a lighter turbidity when compared to the growth control.

Among methods, the strip-disk method appeared to be the most subjective, as zone distortions were sometimes subtle and led to varying interpretations by different technicians (Fig. 2). In contrast, the reading methods for SS and DS align with standard gradient diffusion or disk diffusion guidelines, making them more familiar and straightforward for laboratory technicians. While BDE is not a routine laboratory method, it is relatively easy to interpret, relying on the presence or absence of turbidity as observed by the unaided eye. Although turbidity was occasionally faint or less pronounced than the growth control, technicians were still able to consistently capture results as growth (Fig. 3). Quality control testing demonstrated that most methods had acceptable performance. SS, DS, and strip-disk had some variation of QC results between sites within a method, and this variation of results was also seen in the evaluation results for CA. However, the QC strains for SS, DS, and strip-disk are not standardized as we selected these based on their known phenotype to challenge each method’s ability to interpret susceptibility (ATCC BAA-2146) and resistance (AR Bank AR-0348). Therefore, more robust QC testing is required to determine the optimal organisms for methods where no QC strain recommendations exist. As mentioned before, methodological and analytical differences in protocols and variability in performance even within a single study underscore the need for further studies and standardization of these innovative, yet nuanced, laboratory-developed tests for combination testing, not only for ATM-CZA, but for combination testing more broadly. Therefore, caution should be exercised when implementing these types of tests, as performance varied by method, manufacturer, and investigators. Thus, quality control and thorough validation of such methods are critical to ensure accurate results.

While these methods provide MICs for ATM-CZA and not AZA, the correlation between the MICs of ATM-CZA and AZA was very good, with >97% of MICs appearing within a dilution of each other, and most MICs were exactly the same (>74%). MICs where there was a ±2 dilution difference did not confer any major categorical interpretation differences (e.g., S versus R, R versus S). This suggests that testing ATM-CZA can serve as a reliable surrogate test for AZA MICs. This observation also aligns with other studies that have results for both ATM-CZA and AZA (30).

Our study had several limitations. First, not all isolates were tested with the same reagent manufacturers at both sites due to limited access to certain products and administrative hurdles, like the HiMedia reagents in the United States. Second, results were based on a single replicate, which may not fully capture within-test variability. However, all methods were compared directly to reference broth microdilution by testing all methods on the same day from the same inoculum, supporting the reliability of our performance comparisons. Third, the isolates tested are not reflective of normal patient populations. However, this was an intentional choice for our study to ensure that the methods were evaluated on isolates displaying phenotypes most likely to be considered for ATM-CZA testing. We attempted to test isolates that represent not only susceptible but also resistant strains; however, most resistant strains lingered around the breakpoint, increasing the likelihood of categorical disagreement due to the result’s proximity to the breakpoint rather than a methodological failure. Finally, for SS, we did not calculate the fractional inhibitory concentration (FIC) index. Instead, we relied on the MIC-based categorical interpretations, consistent with conventional gradient diffusion procedures. This decision also ensured we were assessing clinical relevance, as synergy defined only by a fourfold FIC reduction may not always translate to successful clinical outcomes.

MBL-producing Enterobacterales are a global public health concern, as they lack newer effective agents to treat their infections. While cefiderocol, a new beta-lactam agent, is active against carbapenemase-producing gram-negative organisms, including MBLs, recent studies have shown that bacteria harboring MBLs, particularly the New Delhi MBL (NDM), often demonstrate decreased susceptibility to cefiderocol (31–33). In addition, susceptibility testing for cefiderocol has challenges and nuances in both the testing and reading of results (34). The combination of AZA offers a promising therapeutic option for these otherwise hard-to-treat infections. Although AZA has only recently received approval in the U.S. and European markets, laboratories have long been investigating laboratory-developed methods as a surrogate approach to assess AZA activity against MBL-producing Enterobacterales with the use of ATM-CZA prior to availability of AZA. This study evaluated several laboratory-developed methods for testing ATM-CZA that are practical and accessible for routine implementation in clinical microbiology laboratories. Among the four methods tested, we found that most methods and method/manufacturer combinations, except for DS, provided reasonable performance for describing CA. Notably, only BDE exceeded acceptable performance (>90% accuracy), had good reproducibility, and was easy to perform and interpret. As a result, GAIHN-AR participating hospitals in India have implemented BDE for ATM-CZA testing. We also provide the BDE results for the AR Isolate Bank isolates tested for laboratories to use for their own validations (Table S1); furthermore, the AR Isolate Bank has published reference broth microdilution aztreonam/avibactam results for some of their isolates. In addition, these findings support the feasibility of using certain laboratory-developed methods to support clinical decision-making, aiding clinicians when faced with treating infections caused by highly resistant MBL-producing Enterobacterales.

## SUPPLEMENTAL MATERIAL

Table S1 jcm.01800-25-s0001.xlsx List of isolates tested and BDE results for CDC & FDA AR Isolate Bank isolates obtained by both CDC and JHU.

Table S2 jcm.01800-25-s0002.xlsx Performance of four phenotypic methods for aztreonam plus ceftazidime-avibactam AST compared to the reference broth microdilution result after repeat testing.

Table S3 jcm.01800-25-s0003.xlsx Quality control results for reference broth microdilution, broth disk elution, strip stacking, disk staking, and strip to disk proximity methods by manufacturer, site, and quality control organism.

ASM does not own the copyrights to Supplemental Material that may be linked to, or accessed through, an article. The authors have granted ASM a non-exclusive, world-wide license to publish the Supplemental Material files. Please contact the corresponding author directly for reuse.
